# “Reality Made Calibratable”: Walther Moede’s Bimanual Tester, Labor Efficiency, and Psychotechnical “Objectivity” in the Weimar Republic

**DOI:** 10.1007/s00048-025-00425-5

**Published:** 2025-08-20

**Authors:** Agnes Bauer

**Affiliations:** 1 Leibniz Centre for Contemporary History, Am Neuen Markt 1, 14467 Potsdam, Germany; 2 Historical Institute, University of Potsdam, Potsdam, Germany

**Keywords:** Objectivity, Apparatus, Psychology, Weimar Republic, Labor, Material culture, Objektivität, Apparate, Psychologie, Weimarer Republik

## Abstract

This article analyzes how industrial psychologists used mechanical devices to study the efficiency of human labor. One major proponent of this research field, commonly known as “Psychotechnik,” was Walther Moede. He invented a so-called bimanual tester—“Zweihandprüfer”—that enabled him to quantify the subjects’ performance in aptitude tests, and then translate these findings into forecasts of future efficiency and productivity. Industrial psychologists interpreted their results as seemingly objective and unbiased indicators of the subjects’ skills which made it possible to allocate workers, employees, and apprentices to their appropriate position within companies. This article, in contrast, argues that the classification of workers and employees was to a certain degree based on the examiners’ qualitative value judgements. Drawing on printed sources such as psychotechnical journals and textbooks as well as experiments of their own with a bimanual tester, the author is able to show how industrial psychologists interpreted, evaluated, and categorized the participants’ aptitude test results. Psychotechnicians claimed scientific authority and, hence, the power to classify individuals as “failures” or “gifted.” While industrial psychologists argue that they were able to rationalize the distribution of work in the 1920s, this article reveals the leeway there is for interpretation when translating allegedly “objective” test results.

## Abstract (de)

Dieser Artikel analysiert, wie Arbeitspsycholog:innen in der Weimarer Republik menschliche Produktivität mittels mechanischer Apparate untersuchten. Ein Vertreter dieser Forschungsrichtung, die unter dem Namen Psychotechnik bekannt wurde, war der Psychologe Walther Moede. Er erfand den sogenannten Zweihandprüfer, der es ermöglichte, die Fähigkeiten von Proband:innen in Eignungsprüfungen zu quantifizieren. Diese Ergebnisse wurden anschließend in Prognosen zur zukünftigen Produktivität übersetzt. Die Resultate interpretierten Arbeitspsycholog:innen als scheinbar objektives und werturteilsfreies Abbild der jeweiligen Fähigkeiten, anhand derer Angestellte, Arbeiter:innen und Lehrlinge die ihnen entsprechende Position in Unternehmen zugewiesen werden konnte. Dieser Beitrag argumentiert, dass neben den quantitativen Befunden für die Einstufung der Arbeitskräfte auch qualitative Werturteile der Prüferinnen und Prüfer handlungsleitend waren. Auf der Basis psychotechnischer Fachzeitschriften und Handbücher sowie von der Verfasser:in durchgeführter Selbstversuche mit einem Zweihandprüfer wird dargelegt, wie Arbeitsycholog:innen die Ergebnisse der Eignungsprüfungen von Versuchspersonen interpretierten, bewerteten und kategorisierten. Dabei beanspruchten Psychotechniker:innen wissenschaftliche Autorität und wiesen sich selbst die Deutungsmacht zu, die Befähigung von Individuen in Kategorien von „Versagern“ und „Begabten zu unterteilen. Während Arbeitspsycholog:innen für sich beanspruchten, die Verteilung von Arbeitsaufgaben während der 1920er Jahre zu rationalisieren, zeigt dieser Artikel demgegenüber, welche Spielräume bei der Bewertung vermeintlich „objektiver“ Testergebnisse existierten.

## Efficiency and Psychological Instruments: Introduction to an Object-oriented Study

“I would like to emphasize that the practical implementation of psychotechnical work is capable of increasing the joy of work, if we put the right man in the right place, furthermore, that the distrust of the workers has diminished surprisingly quickly, as they have had many opportunities to convince themselves of the objective nature of our testing procedures and their scientific handling.”

„Ich möchte betonen, dass die praktische Durchführung der psychotechnischen Arbeit geeignet ist, die Arbeitsfreude zu erhöhen, wenn wir den richtigen Mann an den richtigen Platz stellen, dass weiter das Misstrauen der Arbeiter überraschend schnell geschwunden ist, da sie sich von der objektiven Natur unserer Prüfungsverfahren und ihrer wissenschaftlichen Handhabung mannigfach selbst zu überzeugen Gelegenheit nahmen.“ (Moede 1919c: folio 104)

**Fig. 1:**
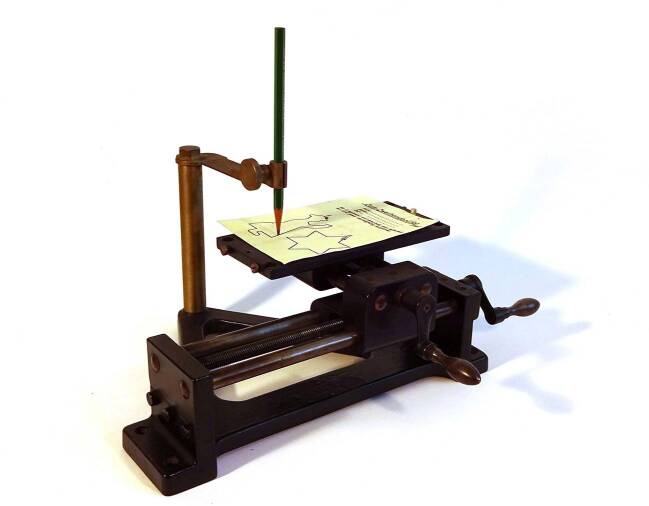
Bimanual tester designed by Moede (30-VIII, Nr. 834), 30 cm width / 22 cm depth / 17 cm height, picture by courtesy of the Psychologiegeschichtliches Forschungsarchiv Hagen (PGFA Hagen)

**Fig. 2:**
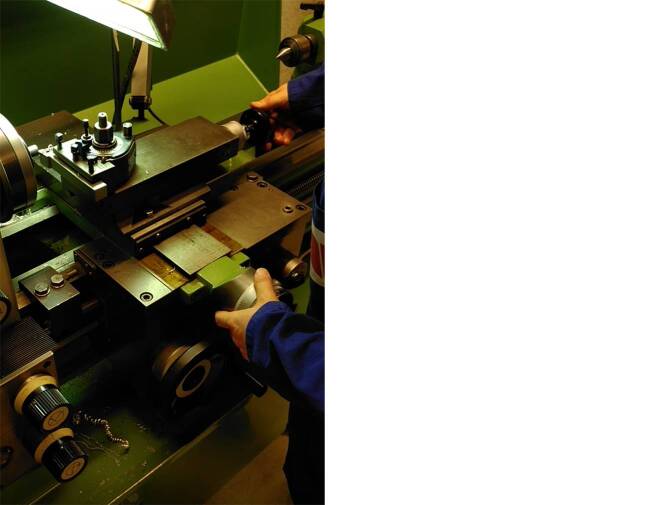
Participant working on a modern turning lathe; picture taken by the author

In writing these lines in a letter to the “Forschungsgesellschaft für wirtschaftlichen Baubetrieb” (research society of efficient construction), the industrial psychologist Walther Moede (1888–1958) hoped to advance a pressing matter. His aim was to rationalize the human body, a goal shared among physiologists and psychologists (Rabinbach 1990: 271). Moede, who coined the term “Wirklichkeit in eichfähiger Form” (reality made calibratable) in 1921 (Moede 1921: 294), participated in the German efficiency movement that treated labor power as a valuable resource which had to be regulated carefully (Meskill 2010: 79). Moreover, the members of the movement believed that it was possible to establish meritocratic principles. Not only national conservative and social democratic politicians but also entrepreneurs embraced this idea in the 1920s. Accordingly, rationalization measures aimed to allocate workers to tasks that matched their abilities and skills. Industrial psychologists or psychotechnicians^1^ like Moede supported this approach by developing aptitude tests. They considered these to be an objective and fair basis of choosing people to hire, in contrast to selection based on familiarity or sympathy. Moede also invented mechanical devices for aptitude tests such as the “Zweihandprüfer” (bimanual tester, or “tester for simultaneous action of both hands”), designed in 1919. It became a prominent instrument that was supposed to quantify the subjects’ efficiency and productivity (see Fig. 1). It was the modified version of a slide rest—a tool common in industrial workshops since the 19th century—that was mounted on turning-lathes and which held the blade to cut and turn metal (Benad-Wagenhoff 1993: 55–56) (see Fig. 2). The bimanual tester was an important instrument in career aptitude tests in Germany during the first half of the 20th century (Zimmermann 1923:68). For instance, during the Weimar Republic, it became a well-established instrument in the technical test arrays of private companies or state-owned enterprises (Moede 1919a; Finder 1923).

Relying on the bimanual tester and Moede’s approach, the article aims to reveal how the bimanual tester was used to select apprentices, workers, and employees based on “objective” criteria: How did Walther Moede use the bimanual tester to determine the manual and cognitive abilities of test subjects? I will also show to what extent his idea of unbiased objectivity fitted into the rationalization movement of the Weimar Republic. The impact of quantitatively measurable test results and subjective judgements in aptitude tests will also be studied: How was Moede able to derive allegedly scientifically objective test results? These findings will be contrasted with a retrospectively replicated test with a bimanual tester. Although the device abstracted from working conditions on the shop floor, I aim to show how industrial psychologists were able to claim that they were nevertheless able to make “reality calibratable.” In particular, I argue that they established practices of simulating, registering, and ranking test subjects’ performance in order to categorize individuals as “failures” or “gifted persons” by their proclaimed scientific expertise.

This article analyzes Walther Moede’s concept of objectivity that not only aimed to rationalize the selection of employees, workers, and apprentices but also to improve productivity during the Weimar Republic. Lorraine Daston and Peter Galison have shown that objectivity is a historical concept that can change depending on scientific traditions (Daston & Galison 2002: 31f.). Accordingly, “mechanical objectivity” predominated from the second half of the 19th century to the beginning of the interwar period and “trained judgement” followed in the subsequent years.^2^ During the transition period after the turn of the century, both ideas of objectivity were in conflict, and converged in the 1920s. (Daston & Galison 2010). This process, I argue, is reflected in the design of Moede’s bimanual tester, which contributed to the emergence to a specific type of objectivity that I term “psychotechnical objectivity.” It claimed unbiased judgement because it made it possible to quantify test results. Moreover, it gave its proponents authority and allowed them to take over aptitude testing from non-trained industrial practitioners.

To link measuring subjects and objectifying results was a common practice among psychotechnicians, shown in their linguistic terms. Moede used the word “eichen” (calibrate). It means to check something for its correct size or weight (“amtlich auf richtiges Maß oder Gewicht prüfen”) (Pfeifer & Braun 1993). Accordingly, industrial psychologists were able to portray themselves as representatives of a hard science (Baumgarten 1928: 156f.; Moede 1927: 347), despite the subjective component—the qualitative assessment—of psychotechnical testing, which will be pointed out in this article.

By presenting an object-oriented study, I complement the historical research of Siegfried Jaeger and Irmingard Staeuble on psychotechnical practices, which has shown how psychology became a politically embedded science. They were among the first psychologists to historically analyze how psychotechnicians claimed that the reason for low productivity was the workers’ personalities (1981). Moreover, they argued that by upholding an image of impartiality, industrial psychology easily put itself into service of the National Socialist regime—as analyzed also by Andreas Killen (Killen 2007).^3^

So far, studies on the history of psychotechnics mostly have taken a bird’s eye view of the practices of how objectivity was established. They study the competences and locations needed for psychotechnical aptitude tests, and illustrate how psychotechnics was part of the 1920s rationalization movement, for example at the German State Railways, the Imperial Post, and in private companies, whose vocational assessment was shaped by psychotechnics (Patzel-Mattern 2010; Gundlach 1993a; Gundlach 1993b; Weber 2001). These perspectives show that psychotechnics was indeed a widespread endeavor, and not a “craze” (Bernet 2016:285; Stadler 2021: 67; Rabinbach 1990:278). Moreover, psychotechnics and modern psychology were also intertwined as, according to Didier Deleule, psychology has been “a seemingly indispensable gear in the social machinery” that tried to find “the identity of a being that can be represented and therefore mastered” by testing human senses (Deleule 2014: 114, 99). Particularly, psychotechnics was part of this regulatory process. It provided a means to measure skills so that, according to test results, individuals could be compared and then allocated to their adequate position in the world of work (Schrage 2001). I would like to add to these important studies how psychotechnical objectivity was established between demands from industrial practitioners and scientific ideals, by looking at the details of translating test results into judgements on individual skill.

The empirical material consists of printed sources, archival material, and a material application of the bimanual tester. The journals *Praktische Psychologie* (1919–1924) and *Industrielle Psychotechnik *(1925–1944) published a wide array of testimonies from industrial practitioners, engineers, and psychologists. I will complement this view with sources from the journal *Psychotechnische Zeitschrift* (1925–1938). Pedagogical journals were also a site for psychological discussions of aptitude (for example the *Zeitschrift für pädagogische Psychologie und Jugendkunde*, on educational psychology and youth studies). Monographs written by psychotechnical experts like Walther Moede himself, but also Franziska Baumgarten-Tramer and Fritz Giese, will complement additional perspectives on psychotechnics. Furthermore, I simulated test practices with one historical bimanual tester, stored in the archive for the history of psychology at the University of Hagen (Psychologiegeschichtliches Forschungsarchiv Hagen—PGFA).^4^ I thus add a material culture perspective to my approach (Gaskell & Carter 2020:2). Investigating material culture means “the study of material to understand culture, to discover the beliefs—the values, ideas, attitudes, and assumptions—of a particular community or society at a given time” (Prown 1993:1). The value in focus here is the psychotechnical scientific idea of objectivity. One possible way to study materiality is RRR—reconstruction, replication, and re-enactment—which is an umbrella term for various similar performative methods that allow, among other things, the exploration of bodily knowledge and sensory skills (Dupré et al. 2020: 9).^5^ Applying the RRR approach on the bimanual tester enabled me to reflect on the stimuli that came from the instrument itself and my surroundings.^6^ This influenced my performance and my inner cognitive and haptic processes that I had not previously been aware of. It made me wonder whether bodily impairment such as bad vision or shaking hands prevented successful performance on the bimanual tester or whether these could be somehow compensated by using clever coping strategies. Hence, using the object puts forward, albeit indirectly, the test subjects’ potential perspective, which is very valuable because in psychotechnical research, testimonies of test subjects are very rare. This indirect perspective led me to the question of whether the bimanual tester actually could simulate the procedure of turning. Replicating the use of the bimanual tester had an orienting function for my work with text sources. Once I paid close attention to sensual phenomena that were conveyed by the object, I then could read the historical written sources with heightened awareness: Did the historical authors refer to similar sensations and what did they conclude from them? Consequently, the article addresses the preoccupation with the embodied knowledge of the human test subjects.

## Historical Background: Efficiency Movement and Psychotechnics from 1900 to the 1920s

The term “Psychotechnik” was first coined by the psychologist William Stern in 1903 and later modified by his colleague Hugo Münsterberg in 1914. Stern differentiated between “Psychotechnik” and “Psychognostik”; the latter providing the means of judging individual values, and the former providing the means to promote desirable behavior (Stern 1903: 28). Münsterberg’s definition was more precise: he labelled psychotechnics as the science of applying psychological knowledge to cultural endeavors (Münsterberg 1920: 1). Münsterberg argued that in particular teachers, advocates, priests, salesmen, politicians, and artists would profit from psychotechnical methods because these would allow them to influence their audience to their benefit (Münsterberg 1920: 7).

World War I is considered the starting point for psychotechnical test procedures in Germany (Jaeger & Staeuble 1981: 67f.). Initially, it provided the methods for testing military personnel, such as experts in sound ranging, or pilots in Germany and Italy between 1915 and 1918 (Wittje 2016: 74, 111; Vinchur 2019: 106–107). It was then that Moede became involved, when he and his colleague Curt Piorkowski tested the aptitude of military car drivers (Gundlach 2019: 31f.). Such tests also flourished in other countries, for example in the USA, where the army started applying intelligence tests in 1917 (Carson 2007). Furthermore, industrial psychology also spread to companies. For instance, women had to pass aptitude tests during World War I, when they were hired by heavy industry or arms factories, replacing conscripted men (Kassel 1997: 33–34; Jaeger & Staeuble 1981: 69f.; Patzel-Mattern 2010: 21–23).

In the 1920s, industrial psychologists narrowed their field of research: they focused mainly on economic and labor contexts in order to improve economic performance, at a time when German industry was ailing in the wake of the defeat in World War I (Patzel-Mattern 2010: 37–40). Psychotechnics thus developed into an important strand of applied psychology that sought to optimize workplace, work techniques, administration (personnel), and also advertising (Moede 1930: 1). Psychotechnical studies, for instance, helped adjust the tools and the setting of the workplace to meet the physical requirements of the workers (Benkert 1925). In the second half of the 20th century, this approach was adopted by ergonomics (Haslacher & Ponstingl 1992). In addition, psychotechnical testing facilitated the selection of personnel because it established procedures for evaluating both workers and employees, which then could be systematically appointed to jobs that matched their skills—a procedure named “Subjektpsychotechnik” (psychotechnics of the subject) (Giese 1925a: 2). Industrial psychologists claimed that their systematic test procedures improved the efficiency of companies, which then would have to deal with less waste and fewer accidents in the workplace, and benefit from higher production rates. Furthermore, guiding laborers to workplaces befitting their ability would bring joy to them, as they would gain a sense of fulfilment from their tasks (Schrage 2001: 81f.). Consequently, “the right man in the right place,” as industrial psychologists and industrial engineers put it, was supposed to bring order and efficiency to the labor market and promised to judge people solely according to their actual aptitude.

Due to its positive effects on performance of the wartime economy, industrial psychology further flourished in the wake of the rationalization movement in the 1920s (Jaeger & Staeuble 1981: 77f.). “Rationalisierung” (rationalization) and its US equivalent “efficiency” remained vague terms that could be interpreted according to one’s economic, political, and social goals. In Germany, “Rationalisierung” referred to “productivity by integration and consolidation, technological modernization and labor process reorganization, the assembly line, and the time-and-motion studies of Frederick W. Taylor” (Nolan 1994: 6). Taylor’s ideas had been discussed in Germany before 1914 (Nolan 1994: 43). However, they gained even more prominence in the 1920s because Taylorism delivered a blueprint for the reintegration of war-disabled men into the labor market (Maier 1970). Georg Schlesinger, engineer and professor for mechanical engineering at the Technical University of Berlin, had already founded a test center for prostheses in 1915 (Harrasser 2013: 103). He was not only an ardent supporter of Taylor’s scientific management—and hence, was nicknamed the “German Taylor” (Rabinbach 1990: 273)—but also his research aimed to improve work processes so as to increase the efficiency of industrial labor. Schlesinger also applied the Taylorist concept of ideal work movements to invalids with prostheses, so that they could fulfil their duties as citizens and remain productive members of society (Horn 2002). It was from Schlesinger’s department that the Institute for Industrial Psychotechnics at the Technical University Berlin-Charlottenburg emerged. In 1918, a research group for psychotechnical experiments that included Walther Moede was established at Schlesinger’s laboratory, which was turned into an institute of the Technical University in 1923 (Spur et al. 2000: 372, 382f.). Moede had been trained as an experimental psychologist by Wilhelm Wundt and Ernst Meumann and was familiar with quantifying tests procedures as early as 1907–1909 (Spur 2008: 24f., 37f.). He continued this scientific tradition during World War I and the interwar period (Ash 2003: 257). Moede hence based his judgement on the test results provided by measuring instruments. Moreover, he had a far-reaching influence on the field of industrial psychology. With Piorkowski, he co-edited the journal *Praktische Psychologie*, which was later renamed *Industrielle Psychotechnik* (Spur 2008: 164–169). In addition, he trained numerous industrial psychologists as an adjunct professor at the Technical University in Berlin (from 1921), and also worked as a lecturer at the Commercial College of Berlin. He remained affiliated with the Technical University until 1946 (Morgenroth 2017: 318–320; Spur 2008: 424). Moede thus left his mark on psychotechnical methods, yet his approach was considered controversial by the renowned psychologists Otto Lipmann and William Stern. While the latter were interested in public vocational guidance, Moede belonged to a group of psychotechnicians that consisted of applied psychologists or trained engineers, who considered themselves psychologically educated industrial practitioners and closely cooperated with private companies.

The practices in industrial psychology were not organized centrally; rather, numerous scientific institutes as well as companies used psychological knowledge to create their own test procedures, adapted to their specific needs. Technical universities and companies were thus fertile ground for applied psychology (Jaeger & Staeuble 1981: 81). In particular, major industrial enterprises, such as AEG, Loewe AG, Siemens, and Osram in Berlin, and Zeiss in Jena, were able to pay for expensive test centers, which were mostly founded shortly after World War I: AEG, Loewe AG, and Zeiss established their test centers in 1918 (Jaeger & Staeuble 1981: 79). Siemens founded its test centers in the early 1920s (Patzel-Mattern 2010: 49). Osram employed an external psychologist, Moede’s colleague Curt Piorkowski, who conducted vocational selection tests in Berlin in 1919 (Weber 2001: 26). According to a survey of the journal *Industrielle Psychotechnik*, 63 test centers existed in German companies in 1926 (Geuter 1984: 221). Mostly, it was companies in the fields of heavy and precision engineering, the electrical and mining industries, and the optical and chemical industries that relied on psychotechnical test centers, which were primarily headed by engineers (Jaeger & Staeuble 1981: 79).

## The Bimanual Tester in the Metal Industry and Walther Moede’s Model of Simulation

The bimanual tester usually consists of a sleigh that can be moved from side to side by rotating a crank on the right, as shown in Fig. 1. On the sleigh, a tray can be moved forward and backwards by rotating the crank on the front side of the device. The tray holds a piece of paper with a printed pattern. A mount holds a pen, pointing down, in an upright position. Users have to rotate the cranks in order to move the tray with the printed pattern underneath the pen in such a manner that they retrace the form without straying too much from it. Instrument firms like Max Marx & Berndt provided those apparatus (Marx & Berndt 1925). The bimanual tester enabled industrial psychologists to examine the skills of applicants for jobs or apprenticeships in metalworking, in which the coordination of both hands was needed, such as turning lathe operators, cutters, or welders (Moede 1919a: 8; Radler 1929).

Usually, engineers and psychologists had at their disposal a whole test battery of different apparatuses and forms. Based on the results, they drew conclusions on the senses, manual dexterity, speed, and attention span of apprentices and job-seeking unskilled workers (Moede 1930: 354, 400). In addition, they assessed the apprentices’ spatial sense, their “practical” or “technical” intelligence, and their ability to carry out assigned tasks. Walther Moede was well aware that manual labor and mind were closely related, and that motor skills were connected with cognitive processes (Moede 1930: 203). But manual skills were easier to assess and quantify (Neumaier 2022). Consequently, Moede emphasized the manual element of the bimanual tester, and claimed that it was by far the best device for quantifying manual coordination skills:

“Apart from the general ability for multitasking, we test the coordination of both hands under specific conditions that are equivalent to the ones in the workshop. There, very often, a bi-manual operation is required, for example when using the slide rest longitudinally and transversely.”^7^

The metal industry was the first site for aptitude tests on apprentices, because it was one of the first industries to have its own apprenticeship programs and no longer relied on traditional handicraft apprentices. Industrial metalworking professions had emerged with industrialization at the turn of the century in the German Empire. Initially, at the end of the 19th century, handicraft had been providing skilled workers who could be employed in industry. Technology, however, advanced rapidly in the fast-growing industry, already shortly after the turn of the century, so that traditionally trained craftsmen and -women could no longer provide the requisite skills (Meskill 2005: 68). Consequently, companies established their own instruction programs and certificates of apprenticeship. Major enterprises like Siemens or Krupp had already established their own training programs before the 1890s. In-house apprenticeships became more common in smaller companies only in and after the 1890s (Meskill 2010: 57). Selecting apprentices was part of a larger process of establishing a professional industrial apprenticeship system, which was strongly pursued by professional organizations such as the German Committee for Technical Schooling (“Deutscher Ausschuss für Technisches Schulwesen,” in short: DATSCH) (Becker 2021: 11). After World War I, apprentices were among the first to be tested with the bimanual tester. Moede’s work on tests for apprentices became the main focus of his Institute of Industrial Psychotechnics at the Technical University in Berlin-Charlottenburg (Haak 1996: 175).

Moede stressed the objectivity of psychotechnical aptitude tests as a fair way of assigning jobs, because he believed that mechanical objects register human performance accurately and without human intervention (Moede 1919a: 81; Moede 1921: 307). Moede was well known for relying on complicated apparatuses that were characterized by their “objective design and mechanization,” as Franziska Baumgarten, a fellow industrial psychologist from Switzerland, concluded in her survey of methods and practices in vocational aptitude tests (Baumgarten 1928: 385–386). Owing to the apparatuses’ mechanical construction, the test subjects’ performance was directly documented. Consequently, these apparatuses were allegedly able to “reduce or even eliminate nearly completely the influence of the test supervisor’s personality,” Baumgarten argued (Baumgarten 1928: 156f.). Even though contemporaries asserted that aptitude tests with Moede’s apparatus were “objective,” from an historical perspective “mechanical objectivity,” as defined by Daston and Galison, was only one part of psychotechnical objectivity. Rather, “trained judgment” was a part of this seemingly mechanical type of objectivity from the start, as the bimanual tester illustrates and as will be discussed here, in the later segments. This trained judgment worked because it could build on the image of mechanical objectivity that Moede created. Moede claimed that simulating industrial work, as with a bimanual tester, would provide objective results that psychotechnicians could translate into forecasts of the subjects’ future efficiency and productivity. In doing so, Moede hoped that he would gain authority and the public would thus trust his approach. This was not an easy endeavor, because Moede had to grapple with two different requirements of testing: the scientific one, which strove for precision and a very clearly demarcated object of study, and the industrial one, which called for practical, cheap, and easy methods.

Moede, who had already designed a test battery for gifted children in 1918, published several programmatic articles on how to conduct a proper aptitude test on apprentices.^8^ He distinguished three categories of test procedure: 1) purely abstract tests in the laboratory, 2) a “scheme of reality” test, in which an apparatus reviews only one work procedure, and 3) the “reality made calibratable” test that enabled a holistic assessment of work performances (Moede 1921: 293). The purely abstract tests could comprise tachistoscopic tests of attention—an ability that was necessary for observing whether a machine ran smoothly—in which test participants were asked to count geometrical shapes or numbers (Moede 1930: 313–315). A typical “scheme of reality” test, according to Moede, allowed for the analysis of specific body functions, for example the sense of touch, which was tested with an apparatus named “Tastsinnprüfer” (tester of tactile sense) (Moede 1920b: 433). “Reality made calibratable” tests enabled psychologists to simulate several steps of a work procedure, such as using tweezers to mount little metal discs on wires, an operation taken from the electrotechnical industry (Moede 1930: 308–311). Moede assigned the bimanual tester to the latter category of performance tests. Most of the test procedures that were categorized in those three groups produced quantitative data: especially, the apparatuses were constructed in such a way that the test subject had to manipulate them to the best of their knowledge, but only the test supervisor could see the hidden scale that showed how much the test subject deviated in their estimation.

Sources indicate that Moede introduced a systematic differentiation of test procedures for a reason. He tried to legitimize his psychotechnical approach as a precise and useful way of selecting personnel for two reasons: firstly, testing applicants as a practice of applied psychology was a new social technique in the early 1920s; and secondly, psychotechnics was criticized from various directions, foremost from philosophy. Applied psychology had roots in experimental psychology, which was considered a rival to philosophy on an academic level. At the beginning of the 20th century, experimental psychologists had to prove that they could contribute to philosophical questions about the human mind (Ash 1995: 8). Moede, in contrast, did not give in to this pressure and limited his research to human qualities that could be measured; he had no aspiration to make statements about the human soul (Rosenberger 2008: 60; Schrage 2001: 100).

Secondly, criticism came from fellow practical psychologists. World War I forced industrial psychologists to introduce aptitude tests without an elaborate theoretical basis, because the need to coordinate assigning soldiers and workers to their work stations was more pressing (Jaeger & Staeuble 1981: 74). Although it was popular in industry, some critical voices in the context of vocational guidance, such as Hellmuth Bogen, the director of the office for vocational guidance in Berlin, cautioned against borrowing new methods that had not yet been fully developed (Weinbrenner 1927: 194). In addition, several applied psychologists such as Fritz Giese or Franziska Baumgarten complained that many devices utilized by engineers without psychological training were actually used only to impress the public, but did not produce viable results (Giese 1925b: 772; Baumgarten 1928: 157). Otto Lipmann and William Stern, both psychologists with an interest in occupational psychology, criticized Moede in 1919 for reducing psychotechnics to a superficial tool for laypeople that lacked a scientific basis (Rosenberger 2008: 59f.). They insisted that only educated psychologists, not hurriedly trained engineers or foremen, who had attended only five-day workshops, should be allowed to conduct aptitude tests. This harsh critique was addressed to Moede, who organized numerous such workshops at his institute in Berlin (Jaeger & Staeuble 1981: 84–85).

By presenting a system of test procedures, Moede tried to prove from the beginning that his approach fulfilled scientific standards, despite the growing criticism from renowned psychologists. This was a strategic move that was supposed to indicate authority in a scientific field.^9^ More generally, it was intended to demonstrate the serious character of the newly established field of industrial psychology and acted thus as a kind of “boundary work” (Gieryn 1983; Wolffram 2012). Boundary work is an “ideological style found in scientists’ attempts to create a public image for science,” one that helps justify claims to authority (Gieryn 1983: 781). It also defended Moede’s understanding of an industrial apparatus-based psychology performed by psychologically educated industrial practitioners against “desktop psychologists,” as he called the faction that insisted on further expanding the theoretical basis for psychotechnics (Moede 1974: 346). In fact, Moede later claimed that Germany relied on a “Metall-und-Eisen-Psychotechnik” (metal and steel psychotechnics), by which he meant that in Germany apparatus-based tests were more widespread than in other countries. Thus, he implied a national style of diagnostics that coincided with his own. Moede contrasted this German model of psychotechnics with other countries, which were more inclined to use paper and pencil tests (Moede n.d.: 1461–1462). At the beginning of the 1920s, in order to prove his diagnostic method was right, Moede relied on quantified test results that had gained a reputation for being neutral and unbiased. Quantification has been used throughout history to stabilize scientific endeavors and show what data is supposed to be relevant and desirable (Porter 1995: 6–8; Mau 2017: 14).

By subsuming the bimanual tester to the test category “reality made calibratable,” Moede implied that he could change the work process of turning on a lathe into a simulation that would produce objective, unbiased data and thus give test subjects faith in the psychotechnical procedures:

“The skilled worker can often be won over by drawing his attention to the calibratable apparatus and making it clear to him that the operations he performs thousands of times on the job or the performance he observes in other workers can be subjected to precise objective evaluation in the laboratory in terms of time and quality.” (Moede 1921: 309)^10^

Moede argued that this kind of testing was a reliable simulation of actual work requirements and that the tester showed exactly that whoever is good at working the cranks of a bimanual tester is also a promising turning lathe operator (Moede 1921: 294). This was not trivial. Industrial psychologists were not at all sure to which degree specific production steps could be abstracted and made into test procedures without becoming invalid (Sachs 1925: 101f.). However, Moede was positive that he could project reality onto a mechanical object.

## The Bimanual Tester: Simulating Working Conditions of a Slide Rest? An Experiment of My Own

Simulating the “reality” of working on a turning lathe equipped with a slide rest was the first operational step that the bimanual tester was supposed to fulfil. In the 1920s, it was an essential epistemological question whether simulating practical work from the factory in a laboratory-like setting would produce reliable data for measuring and evaluating human skills.

The historical records show that the bimanual tester was used to test the coordination of both hands. Consequently, the bimanual tester was supposed to simulate the manual operation of a slide rest. The historical research argues that working on a slide rest required geometric understanding (Benad-Wagenhoff 1993: 164). In order to see whether there might have been even more implicit characteristics, I reconstructed the test person’s impression by working with both a slide rest and a historical bimanual tester and by comparing the body movements required by the two devices.

In February 2020, I participated with fellow students in a metal workshop at the Technische Universität Berlin, where I was trained in working with a turning lathe. We used it for cutting an aluminum piece shown on the left edge of Fig. 2, by steering the blade towards the work piece which was fixed in the chucking element of the turning lathe. Operating a turning lathe for the first time can be confusing. A beginner has to figure out the rotational directions of the cranks moving the tool either from the left to the right or back and forth. This figuring-out turns out to be a cognitive challenge: the operator does not hold the tool in his or her hand and does not steer the tool directly, but via the machine—an example for a machine tool technology (Benad-Wagenhoff et al. 1993: 193). Instead, the figuring-out is like a cognitive sticking point that needs to be resolved. Understanding how the slide rest is constructed might actually help overcome this hurdle: to know that the crank at the front connects to an axis along which the tool goes back and forth, or to know that the crank to the right connects to a left-right-axis.

Initially, the participants in the workshop hardly ever used both hands when working on the turning lathe; they rotated the cranks one after the other, not at the same time. After a few tries, the participants felt more familiar with the slide rest and the aforementioned cognitive sticking point slowly faded into embodied knowledge. That is an observation I made as I was watching the other participants in the workshop and which I tried to understand by asking the participants to describe the sensation they felt, after they were done operating the turning lathe.

Apart from mastering the steering, a couple of sensual impressions contributed significant feedback for working on a slide rest: the connection of tool and workpiece resulted in a certain amount of resistance, accompanied by a specific noise—more power had to be applied to rotate the cranks, when the chisel cuts into the material. Working on a turning lathe thus involves more than just the visual sense.

A couple of points need to be considered when reconstructing historical processes. First of all, the other participants and I were complete beginners. A beginner cannot be compared to a skilled metal turner. Second, we used a modern turning lathe. The technical development of turning lathes in the 20th century has seen a few innovations, mainly in regard to special machinery, higher rotational speed, and harder material for blade edges (Benad-Wagenhoff et al. 1993: 228–30). However, the steering element has remained the same on turning lathes that do not work with computer numeric control (CNC). Keeping those two mitigating factors in mind, it still seems plausible that several key elements of operating a slide rest were illustrated: Key elements were 1) cognitively understanding the movements of the slide rest, 2) connecting to the control element, not necessarily to the tool, and 3) having accurate vision, coordination, and hearing, in order to not mess up the steering and ruin both tool and workpiece.

**Fig. 3:**
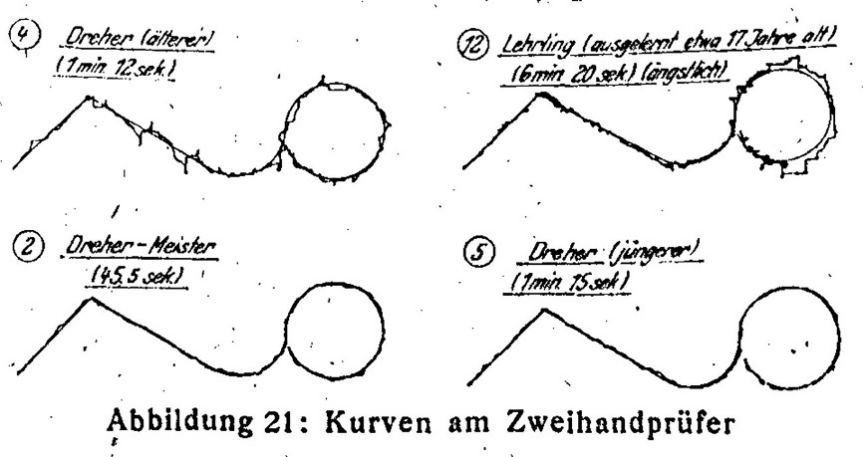
Examples of curves produced on the bimanual tester; Moede 1919a:71

In Hagen, I compared the experiments from the workshop with operating a bimanual tester, which resulted in ambivalent impressions. Using the bimanual tester, the same cognitive barrier had to be overcome, especially when I had to decide which crank and which sense of rotation resulted in which direction of the tray. The most striking difference was that actually both hands were supposed to be moved at the same time for completing inclined and curved lines. The paper slip that came with the bimanual tester in Hagen showed two figures: a human form and a star which you can see in Fig. 1. In fact, this form was usually drawn with a slightly different version of the bimanual tester, which fulfilled the same function, and which was designed by Hans Rupp, who tested apprentices at Siemens-Schuckert (Rupp 1925). In contrast, the original design of the paper template by Moede showed a line in an inclined and curved form, which you can see in Fig. 3 (Moede 1919a: 71). Horizontal or vertical lines are easy to draw on Moede’s bimanual tester because only one crank is needed to move the tray. It was the inclined lines that required the use of both hands at the same time. However, the angle in the star form was not exactly 45 degrees, so that my hands could not go at the same speed when rotating the cranks. This was confusing at first. But the most critical spots were the points of the stars, where I had to change the direction of the crank rotation and to try and remember which hand movement was correct. If I had used the original print design by Moede, it would have been even more challenging: first, both hands then have to rotate at the same speed for an incline of 45 degrees, then they have to change direction and vary the rotational speed of both hands asymmetrically for the declining line that turns into a curve.

In addition, the kinesthetic experience varied: using the bimanual tester was much lighter work; drawing a pencil line presented less drag than actually chipping aluminum. However, it was more difficult to see the trace of the pen and whether it actually touched the printed line because the pen was held in a vertical position and hence blocked the vision. With a turning lathe’s chisel, the tool is held horizontally and thus allows a better view of the work piece. That is why using the bimanual tester felt at the same time playful and slightly irritating. Besides, the bimanual tester did not simulate moving the work tool, but actually the workpiece. In fact, historical sources show that these problems were addressed only in the late 1930s, when an altered version of a bimanual tester with a moving work tool was used in psychological research (Flesch 1937). Moede did not seem to have reflected on this problem.

As a result, working with a slide rest was a matter of steering and understanding the movement of the work tool on a two-dimensional coordinate plane. The bimanual tester was able to simulate this characteristic. However, the bimanual tester made high demands on the test person’s coordination skill by requiring inclined or even curved lines right from the start. In addition, the lack of a sensual feedback such as the material resistance of the workpiece against the tool or the noise of the cutting tool or the turning lathe’s motor seems striking, as these pieces of sensual information assert whether the turner is operating the machine properly. The bimanual tester’s sensual feedback is only visual.

Again, mitigating factors need to be considered. I wondered whether using a curved line on the bimanual tester might have been too demanding for beginners and consequently implied that turning curves might have not even been a skill that was necessary for turning. The slide rest was introduced mainly for one reason, namely to make parallel or rectangular outlines more precise. So, turning curves on it was theoretically possible, but did not gain the benefits a slide rest presented (Benad-Wagenhoff 1993: 57f.). However, Fig. 3 shows that adept turners were in fact able to turn curves perfectly, whereas the apprentice who is labelled as a 17-year-old “fearful” person produced more of an angular line than a curved one. Statements made by historical test participants are hardly to be found in sources of the 1920s. Only in the late 1930s did one study actually inquire into the test participants’ kinesthetic experiences while using the bimanual tester (Flesch 1937).

Still, the experimental use of both a turning lathe and the bimanual tester made me realize that certain sources of sensual feedback were purged in this allegedly exact simulation of a turner’s basic work operation. This leads to the question of whether the bimanual tester produced reliable data for predicting who is skilled enough for manual labor. Historical records show that beside the actual apparatus, there was a certain number of testing practices involved in order to fill this interpretational gap between simulating working conditions with a bimanual tester and its model—the slide rest.

## Oscillating Between Objectivity and Subjectivity: Practices of Registering and Ranking

Registration, that is, documenting the test subject’s actions on the bimanual tester, was the second analytical step that was closely intertwined with the third step of ranking the test results. Even though each step can be distinguished analytically, they both merged in the test procedures. According to contemporary researchers, scientific apparatuses made it possible to count and measure either the time that had passed before a test had been completed or the number of errors that the test subject had made. They would thus guarantee exact and objective test results, by showing that “for each apparatus the test subject’s performance is clearly and justly summarized into a single number,” as one of Moede’s co-workers, Bernhard Herwig, framed it (Herwig 1920–21: 51). The bimanual tester, however, neither automatically counted errors nor measured the test time. Thus, the psychologist clocked the time and gauged the pencil line on the paper template that had been produced by the test subject. In some cases, the actual test was conducted by an assistant. In the 1920s, there was no standard procedure such as evaluation templates that allowed for the identification of errors; rather, the psychologists had to resort to their individual judgement. Consequently, the bimanual tester did not produce results of “mechanical objectivity,” but had to rely on human interpretation.

In order to rule out subjectivity, psychologists and engineers experimented with different variants of the bimanual tester in their psychotechnical institutes: there was Fritz Giese at the Technical University of Stuttgart, Bernhard Herwig at Moede’s Institute for Industrial Psychotechnics in Berlin-Charlottenburg, or the railway engineer Finder at the *Psychotechnische Versuchsstelle* (psychotechnical test center) of the German State Railway in Berlin in the early 1920s. For instance, one variation came with a metal template which left only a narrow slit for the pen to move in (Giese 1925b: 206). The participants were supposed to move the tray so that the pen did not touch the sides of the slit. If they did not succeed, an electrical circuit was closed, which counted an error (Herwig 1922: 128). Another electrical registration allowed participants to stray off, but the more they deviated, the more different electrical contacts were triggered (Finder 1923: 360). Adding this electromechanical application to the analogue bimanual tester, the psychologists hoped to create a procedure for quantitatively registering the test subject’s operation. However, even those technical improvements did not allow for a clear identification of errors. When participants did not use both hands simultaneously to draw a straight inclined line, but created little “stairs” by rotating the cranks alternately, they did not necessarily deviate so much from the optimal inclined line that an electric contact was triggered. The psychologists were very well aware of such strategies because they closely monitored how the test subjects tried to pass the test. Even though subjects might have passed the test, psychologists nevertheless considered inclined lines with “stairs” as a product of cheating and, hence, as a poor performance; a practice that existed well into the 1930s (Herwig 1922: 128f.; Dilger 1934). Another downside of test devices were costs (Finder 1923: 360). The price of the analogue bimanual tester ranged from 160 Marks in 1925 to 106 Marks in 1927 (Marx & Berndt 1925; Weinbrenner 1927). For comparison, the average monthly pay for a laborer in 1925 was 140 Marks (Statistisches Bundesamt 1987: 34). It seems likely that a bimanual tester with an electrical circuit was even more expensive and thus could not be afforded by small test centers. Due to both constraints, a pure quantification of tests with the bimanual tester did not occur in the 1920s.

Instead, after having clocked the time, psychologists compared the individual curves on paper slips with given samples that had been classified into five grades (Baumgarten 1928: 193). Sources do not reveal whether each test center produced its own set of samples by conducting a pre-test on a group of volunteers or whether there was a standard set of samples that had been produced by one or several test centers and then been distributed among them. In any case, the samples from the pre-test were sorted according to aesthetic criteria like the smoothness of the line. They were divided into five or fewer grades. For each grade, one characteristic curve was chosen from the sample. Based on this curve, the psychologists ranked the actual test results (Baumgarten 1928: 193). They considered the elapsed time less relevant for the final assessment than the aesthetic appearance of the curve (Hamburger 1921: 58). Furthermore, the ranking of the curve’s appearance was based solely on the investigator’s experience (Herwig 1922: 129). Subjective criteria thereby entered into test procedures that were intended to objectively describe the skills of applicants, workers, and employees. Franziska Baumgarten criticized this approach as “arbitrary and subjective” in a twofold manner already in 1928 (Baumgarten 1928: 193).

However, the subjective assessment of industrial psychologists was deemed sufficient to form an appropriate opinion about the test subject’s performance. The state railway engineer S. Finder concluded that the judgement of psychotechnical experts mostly coincided with the results from the electromechanical bimanual tester, which was built at the psychotechnical laboratory of the German state railway (Finder 1923: 360). Moede’s coworker Richard Hamburger, a trained engineer, was even skeptical of assessing the curve quantitatively: “To judge the quality of single performances by numbers is a daring endeavor with subjective, inevitable additives. Looking at the curves however shows directly and clearly, that some errors that hinder participants from solving the task remain until the end” (Hamburger 1921: 59).

Not only with regard to the bimanual tester, but in general, the subjective judgement was considered a sometimes necessary, quick, and easy way to assess performance in a test, particularly in industrial environments of practical work (Rupp 1925: 22). Especially when the participants were observed while doing a test, “one may rely on the experienced psychologist unflustered; he will rarely err” (Weber 1927: 384). By relying on their “trained judgment,” industrial psychologists sought to distinguish the “gifted” from the “failures,” or, as Hans Rupp put it in 1925: “Practical law has it, at least for mass production, it is more important to eliminate the ‘bucks,’ than to court the ‘artists.’”^11^ The knowledge of how to judge test subjects properly was considered an expertise of psychologists—foremen and master craftsmen who did not have any psychological training were deemed unreliable judges by most industrial psychologists. Theodor Valentiner, a psychologist working with adolescents, thought practitioners were too personally involved with the apprentices and workers and evaluated these on the basis of an allegedly incorrect gut feeling (Valentiner 1922: 14). Hildegard Sachs, a psychologist who concentrated on vocational guidance, claimed: “The practitioner differentiates according to effective performance and—sympathy” (Sachs 1925: 105).^12^ The foremen’s assessment could never be perfect, concluded psychologists Bramesfeld and Eberle in a study on “The psychology of the practitioner’s judgement” (Bramesfeld & Eberle 1927: 306). At the very least it had to be complemented by the psychologists’ ostensibly more legitimized expertise.

To conclude, the bimanual tester allowed for three steps of operation: the simulation of steering a slide rest; the registration of the test subject’s operation in the form of a pencil line; and the ranking of this pencil line by means of aesthetic criteria. The bimanual tester thus was a tool of selectively simulating reality: on the one hand it appeared as a device that seemed to guarantee mechanical objectivity; on the other hand the test subjects’ skills were, nevertheless, in fact, subjectively assessed by industrial psychologists on the basis of the latter’s “trained judgment.” The bimanual tester was not the only qualitative test procedure allotted in Moede’s test arrays. Especially tests that analyzed the dexterity of apprentices involved methods such as bending wires or making cut-outs, which could not be quantified but had to rely on qualitative assessment by the test conductor (Moede 1930: 356, 358). The bimanual tester, however, was a unique example of a seemingly mechanical device that still produced subjectively assessed results, which shows how a mechanical appearance lent itself to an image of mechanical objectivity, although its function was in fact more differentiated.

## Conclusion: Psychotechnical Authority and Testing as Selecting

The 1920s were a time of intensified rationalization, during which applied psychology was able to flourish in Germany. This psychological field was known for its extensive use of test apparatuses (Herwig 1922: 115; Baumgarten 1928: 157; van Strien 1997). It also presented an opportunity for academic psychologists, engineers, businessmen, and educators alike to share psychological test practices that were used in aptitude tests. Walther Moede was among the first psychotechnical experts to influence the test designs used in those aptitude tests.

Moede used the bimanual tester to qualitatively determine the manual and cognitive abilities of his test subjects, who were apprentices and workers in the metal industry. By the three steps of simulating, registering, and ranking, industrial psychologists like Moede assessed the subjects’ ability to coordinate both hands at the bimanual tester. They did so by sorting the test results based on both previously determined characteristics of ideal performance curves and their own experience. This registration was then translated into a normative assessment of “good” or “poor” performance and hence made it possible to establish seemingly objective criteria on the candidates’ quality. For industrial psychologists and industrial practitioners, the pragmatic goal of such a ranking procedure was to effectively, that is, quickly, select “gifted persons” and exclude “failures.”

This qualitative approach seems all the more interesting because Moede propagated a psychotechnical objectivity that was allegedly based on unbiased judgement. This kind of objectivity fit well into the rationalization movement of the Weimar Republic, because freedom from value judgements was seen as the quintessence of the German aptitude tests, which were an important part of the efficiency movement. Work forces were to no longer be picked and chosen based on sympathy, but only based on their performance. Furthermore, an integral part of the efficiency movement was that it should be accompanied and enforced by scientific measures. This benefited industrial psychologists such as Moede, who claimed to have the true expertise in assessment and took over the evaluation from industrial foremen and industrial master craftsmen without psychological training, who were said to be too emotionally involved.

However, quantitatively measurable test results and subjective judgements were intertwined in aptitude tests. Although Moede relied on complicated apparatuses that promised quantitative test results, in some cases, as with the bimanual tester, qualitative assessment was accepted, if not preferred over quantitative data. The industrial psychologists used the seemingly unbiased character of the quantitative, mechanical apparatus in order to gain an image of psychotechnical objectivity. This image remained even though the industrial psychologists resorted to qualitative assessment, too, which they justified with their “trained judgment” as psychologists.

Moede made sure to present a systematic order of diagnostic methods, to highlight the scientific character of his applied psychology; and he promised to simulate reality as truly as possible in order to gain objective results from his tests. He coined three categories in the simulation of work operations: the most abstract one was laboratory tests, the less abstract one was schemes of reality, while “reality made calibratable” was supposed to simulate a work activity in the least abstract way. The bimanual tester was an example of reality made calibratable, which meant that Moede had to abstract the practice of turning to a certain extent, but tried to stay as true to reality as possible.

However, Moede had to fulfil demands both from a scientific and an industrial-practical point of view. He had to make the aptitude tests as precise and measurable as possible, but he could not make the aptitude test too abstract, either. Otherwise, the test subjects would have refused to take the tests. In some way, the bimanual tester offered a different experience than a turning lathe, because it purged the activity of turning of certain sensual impressions. The cognitive challenge remained. However, the replication showed that in the 1920s, industrial psychologists decided to dispense with some aspects of the original work activity (moving the tool, the horizontal position of the tool, drag from cutting into the workpiece, sound, vibration), which showed that designing a test allowed space for the industrial psychologists’ discretion. Ultimately, this translation of the slide rest’s function from workshop to laboratory could not fulfil Moede’s expectation, because simulating a work environment was far too complex.

Although the psychotechnical objectivity in focus here is unbiased judgement, subjective assessment on the bimanual tester remained in practice and did not challenge the psychologists’ own impartiality. This favored entitling and excluding test takers to or from work or apprenticeships along socially constructed lines that were drawn on the psychologists’ authority. The test-conducting psychologist decided whose line was “smooth” or pretty enough to be considered the work of a potential apprentice. This argument is all the more compelling in cases where the psychologist did not work with anonymized test results. After 1933, many applied psychologists such as Walther Moede tried to adapt their work to the ideological goals of the Nazi regime (Moede et al. 1933). The tests that relied on a psychologist’s trained judgement remained in use, for example for selecting forced laborers in industrial work (Schorn 1942). This opened up a new chapter in the history of industrial psychology in Germany, because assessment no longer involved performance alone, but also, increasingly, biologistic and racist attributions (Geuter 1984; Raehlmann 2005).

## Footnotes

1 “Psychotechnik” or “Industrielle Psychotechnik” is a historical term that was and is still often used synonymously with industrial psychology (Geuter 1984: 212f.; Vinchur 2019: 2). Other similar historical terms were practical psychology (“praktische Psychologie”) or psychology of economics (“Wirtschaftspsychologie”). This wide array of terms shows how vaguely this early field of applied psychology was defined. For clarification, whenever the adjective “psychotechnical” is used in this paper, it refers to industrial psychology in Germany.

2 Mechanical objectivity is “the insistent drive to repress the willful intervention of the artist-author, and to put in its stead a set of procedures that would, as it were, move nature to the page through a strict protocol, if not automatically” (Daston & Galison 2010: 121). Trained judgment, in contrast, is the skill of “seeing scientifically through an interpretive eye,” which is the result of training and experience (Daston & Galison 2010: 311, 313).

3 A broader perspective on psychology in general is presented for example by Schönpflug (2017).

4 A catalogue of available historical instruments can be accessed online: Bettenhaus (2016).

5 For examples, cf. Fickers (2013:273); Fickers (2015); Sibum (2022); Heering (2008); Voskuhl (1997).

6 The historical practices related to scientific instruments and experiments have been objects of study since the 1980s (Baird 2004; Bourguet et al. 2002; Schmidgen 2000; Schmidgen 2008; Schmidgen 2015; Shapin & Schaffer 1985).

7 The original quote says: “Neben der allgemeinen Befähigung zur Mehrfachhandlung prüfen wir das Zusammenarbeiten zweier Hände unter besonderen Bedingungen, wie sie der Werkstättenarbeit entsprechen. Dort ist sehr oft Betätigung der Doppelhand erforderlich, beispielsweise bei Bedienung des Längs- und Quersupports” (Moede 1919a: 71).

8 Cf. Moede and Piorkowski (1918); Moede (1919a); Moede (1919b); Moede (1919–20); Moede (1920a).

9 This strategy was used on several occasions in the history of science. Cf. Van Wyhe (2002).

10 The original quote says: “Der Facharbeiter kann oftmals dadurch gewonnen werden, daß man ihn auf die eichfähige Apparatur hinweist und ihm klarmacht, daß die von ihm im Beruf tausendmal ausgeübten Griffe oder die von ihm an anderen Arbeitern beobachteten Leistungen im Laboratorium einer genauen objektiven Auswertung nach Zeit und Qualität unterzogen werden können”; Moede (1921: 309).

11 The original quote says: “Das Gesetz besagt für die Praxis, wenigstens für Massenarbeiten, daß es wichtiger ist, die ‘Böcke’ auszuscheiden, als ‘Künstler’ zu werben”; Rupp (1925: 74).

12 The original quote says: “Der Praktiker aber unterscheidet nach effektiven Leistungen und – nach Sympathien”; Sachs (1925: 105).
